# Prevalence and risk factors of drug-related problems identified in pharmacy-based medication reviews

**DOI:** 10.1007/s11096-020-00976-8

**Published:** 2020-02-05

**Authors:** Raphael Sell, Marion Schaefer

**Affiliations:** Charité – Universitätsmedizin Berlin, corporate member of Freie Universität Berlin, Humboldt-Universität zu Berlin, and Berlin Institute of Health, Institut für Klinische Pharmakologie, Charitéplatz 1, 10117 Berlin, Germany

## Abstract

*Background* Medication safety is a major health concern, especially for older patients, in whom drug-related problems occur frequently as a consequence of polypharmacy and frailty, increasing the risk of adverse drug events. *Objective* To investigate the prevalence and types of drug-related problems in community pharmacies and to identify associated risk factors in order to adjust the focus of care. *Setting* 300 German community pharmacies in Saxony-Anhalt (Germany). *Method* In April 2015, community pharmacists conducted brown bag medication reviews for primary care patients, in which they identified and solved drug-related problems with patients or their physicians. Data from these reviews were analyzed, including frequency and nature of problems and their respective resolutions. Potentially inappropriate medications according to the PRISCUS list were identified by post hoc analysis. Risk factors for drug-related problems were determined using bivariate and multivariate logistic regression analysis. *Main outcome measure* Prevalence and risk factors of drug-related problems. *Results* 1090 medication reviews were conducted. On average, patients were 72.0 ± 9.1 years old and had 10.6 ± 3.7 medications, 62.0% (n = 676) presented a medication plan. Knowledge gaps about medications were detected in almost a third of patients (n = 345). Drug-related problems were identified in 84.2% (n = 918) of patients (in 3836 medications). Frequent problems concerned drug–drug-interactions (53.7%, n = 585) as well as drug use and adherence (46.7%, n = 509). Most problems (72.2%, n = 2769) were resolved between pharmacist and patient. Knowledge gaps and the number of drugs were independently associated with a higher risk of drug-related problems. For older patients, potentially inappropriate medications were a risk factor in bivariate, but not in multivariate analysis. *Conclusion* Pharmacists identified and resolved considerable rates of drug-related problems, suggesting that they are capable and well-positioned to conduct medication reviews. Knowledge gaps, the number of drugs, patient age and, in older patients, potentially inappropriate medications may indicate an increased risk for drug-related problems.

## Impacts on practice

- Pharmacy-based medication reviews are a feasible way to identify and resolve drug-related problems.
- Incorporating risk factors for drug-related problems may facilitate efficient selection of patients who benefit from medication reviews.
- Knowledge gaps increase the risk of drug-related problems, thus informing patients about drug indications should be considered an essential part of medication reviews.

## Introduction

Drug-related problems (DRPs) are defined as “events or circumstances involving drug therapy that actually or potentially interfere with desired health outcomes” [1]. This includes adverse drug events (ADEs), which have been judged preventable in 11% to 38% of cases [2]. As many as 28% of all emergency department visits are drug-related [3]. The yearly costs of drug-related morbidity and mortality in the United States have been estimated to exceed $177 billion [4]. Thus, to resolve DRPs is essential to improve medication safety.

Older patients are at particular risk of suffering ADEs, due to multimorbidity, polypharmacy and frailty [5, 6]. Potentially inappropriate medications (PIMs) have been associated with increased rates of ADEs and hospitalizations in the ever-growing senior population [7–9]. Several PIM criteria have been conceived to account for cross-national differences in pharmacotherapy [10]. The German PRISCUS list does not necessitate clinical data [11], rendering it useful in pharmacy practice. We thus preferred it over tools which require diagnoses or laboratory values (e.g. Medication appropriateness index, STOPP criteria and FORTA list) [12–14].

Although pharmacy-based medication reviews provide a systematic means to identify and resolve DRPs, they are still rare in routine care in Germany [15]. During the dispensing process, DRP detection is restricted to drugs dispensed at that time [16–19]. However, adequate identification of problems involving multiple medications, e.g. drug–drug-interactions, requires complete medication lists, furthermore, time for analysis is limited. A more comprehensive approach is the type 2a medication review [20], also known as *brown* *bag* medication review [21], in which patients bring their complete medication to a pharmacy. By this method, pharmacists detected higher rates of DRPs [22]. In several countries, pharmacist-led medication reviews have been shown to improve a variety of patient-relevant outcomes [23].

Presently, research on medication reviews in community pharmacies is still limited. Risk factors for DRPs, particularly the influence of PIMs, remain to be determined.

## Aim of the study

This study investigates the prevalence and types of DRPs in patients undergoing medication review in community pharmacies, as well as the rate of PIMs in a subgroup of older patients. Furthermore, it determines potential risk factors for DRPs.

## Ethics approval

In this study, we retrospectively analyzed anonymized data from medication reviews performed for consenting patients. Reviews were conducted as part of pharmacists’ care without external intervention. Both patients and pharmacies were anonymized, no demographic data other than age and sex were collected and solely aggregate data are presented. Therefore, institutional review board approval was waived.

## Method

### Study design

This cross-sectional study analyzed data collected in a project promoting medication safety by the Chamber of Pharmacists of Saxony-Anhalt (Germany) in April 2015. All 612 local community pharmacies were asked to conduct brown bag medication reviews. Pharmacists participated in the study voluntarily and without remuneration. Without predefined inclusion criteria, they invited up to five patients to participate in guideline-based medication reviews [24]. In short, patients brought all their medication to appointments, where pharmacists performed medication anamnesis and collected additional information. Subsequently, the pharmacists reviewed the medication, identified DRPs and developed recommendations. At a second appointment, these were presented to patients and integrated into therapy. Where it was deemed necessary and consented to by patients, their physicians were contacted to facilitate DRP resolution.

### Data collection

Pharmacists documented anonymized data using structured sheets, which contained each patient’s age, sex, possession of a medication plan and a comprehensive medication list. This list included drug name, active substance, strength, formulation, prescription status, dosage regimen and current use. Furthermore, pharmacists documented knowledge gaps (i.e., patient uses a drug without knowing its indication), identified DRPs according to predefined categories and means of resolution (with patient, with physician, not resolved). Finally, net review duration (i.e., the added duration of first appointment, medication review and second appointment) was documented. Completed sheets were collected and inserted into a database. To facilitate further analysis, we coded drugs according to the anatomical therapeutic chemical (ATC) classification [25] and problems according to the PI-Doc^®^-classification [26], which had been used for DRP documentation earlier [18, 27]. The results presented here have not been reported before.

### Subgroup analysis in older patients

In a post hoc analysis including patients over 65 years, we identified PIMs according to the German PRISCUS list [11], which contains 83 drugs judged potentially inappropriate for older patients. For eleven drugs, inappropriateness depends on either daily dose (e.g. zolpidem ≥ 5 mg/d) or drug formulation (e.g. immediate-release nifedipine). To prevent overestimation, such cases were only considered PIMs if the specific daily dose or drug formulation was judged inappropriate.

### Statistical analysis

For continuous variables, mean and standard deviation (SD) or median and interquartile range (IQR) are given; for categorical variables, absolute and relative frequencies are given. Student’s *t* test for independent samples was applied to analyze differences in continuous variables. Pearson’s correlation analysis was performed to investigate correlations between continuous variables. Pearson’s Chi squared test was applied to analyze relationships between categorical variables.

Determinants for DRP presence were identified by logistic regression analyses. Therein, DRP presence was considered the dependent variable, while age, sex, medication plan, knowledge gaps and the number of drugs were considered independent variables. First, bivariate analyses were performed separately for each independent variable. Second, all independent variables were included into multivariate analysis to control for confounding factors and calculate adjusted odds ratios. Finally, the analyses were repeated for older patients with the added independent variable PIM use.

Cases with missing values were excluded from relevant analyses only (pairwise exclusion). All tests were performed two-sided, findings with *p* < 0.05 were deemed statistically significant. Odds ratios (OR) and 95% confidence intervals (CI) were calculated. Data processing and post hoc-coding were performed using Microsoft^®^ Excel^®^ Version 2010. Statistical analyses were performed using IBM^®^ SPSS Statistics^®^ Version 24.

## Results

### Patient characteristics

Pharmacists in 300 pharmacies conducted medication reviews for 1090 patients; 51.9% were female and 62.0% had medication plans (Table 1). In total, patients used 11,579 drugs, resulting in 10.6 ± 3.7 drugs per patient (median: 10, IQR 8–13). More drugs were used by women (10.9 vs. 10.3, *p* = 0.017) and patients with medication plans (10.8 vs. 10.2, *p* = 0.014). Furthermore, the number of drugs correlated with patient age (r = 0.131, *p* < 0.001, *df* = 1051). On average, patients used 9.2 ± 3.2 drugs regularly and 1.5 ± 1.7 drugs as needed. Polypharmacy, defined as regular use of five or more drugs, was present in 1052 patients (97.1%).One-fifth of medications (n = 2325) were available over-the-counter (OTC). According to patients, 1177 drugs (10.2%) were not in use at the time of review.

**Table 1:** Patient characteristics

	All patients^a^ (n = 1090)	Subgroup ≥ 65 years^a^ (n = 830)
Age (years)		
< 65	223 (20.5%)	0
65–69	134 (12.3%)	134 (16.1%)
70–74	226 (20.7%)	226 (27.2%)
75–79	271 (24.9%)	271 (32.7%)
80–84	126 (11.6%)	126 (15.2%)
≥ 85	73 (6.7%)	73 (8.8%)
Missing data	37 (3.4%)	0
Mean ± SD	72.0 ± 9.1	75.6 ± 5.8
Sex		
Male	523 (48.0%)	387 (46.6%)
Female	566 (51.9%)	443 (53.4%)
Missing data	1 (0.1%)	0
Medication plan		
Without plan	366 (33.6%)	265 (31.9%)
With plan	676 (62.0%)	532 (64.1%)
Missing data	48 (4.4%)	33 (4.0%)
Knowledge gaps		
Without knowledge gaps	745 (68.3%)	547 (65.9%)
With knowledge gaps	345 (31.7%)	283 (34.1%)
Number of drugs^b^		
< 10	476 (43.7%)	355 (42.8%)
10–14	450 (41.3%)	344 (41.4%)
≥ 15	164 (15.0%)	131 (15.8%)
Mean ± SD	10.6 ± 3.7	10.7 ± 3.7

^a^Percentages are column proportions

^b^No group was created for patients with < 5 drugs, because few patients fulfilled this criterion

Knowledge gaps were detected in almost a third of patients (n = 345/1090), the proportion increased steadily with age from 23.8% (n = 53/223) under 65 years to 39.7% (n = 29/73) over 85 years (*p* = 0.001). Knowledge gaps were more common among men (35.8%, n = 187/523) than women (27.9%, n = 158/566; OR 1.281, 95%-CI 1.075–1.526, *p* = 0.005), whereas medication plans had no effect (*p* = 0.658). They were less common for OTC drugs (OR 0.636, 95%-CI 0.525–0.770, *p* < 0.001). Drugs with knowledge gaps (n = 930, 7.9%) were more often involved in DRPs (OR 1.348, 95%-CI 1.174–1.548, *p* < 0.001).

### Potentially inappropriate medication in older patients

Among patients over 65 years (n = 830/1090), we identified 247 PIMs in 202 patients (24.3%). Most of these patients (n = 161) used one PIM, while 37 patients used two PIMs and 4 patients used three PIMs concurrently. As Table 2 shows, PIM prevalence was comparable across age groups, but significantly higher among women (OR 1.672, 95%-CI 1.207–2.315, *p* = 0.002). Patients with PIMs used more drugs; PIM and drug numbers correlated (r = 0.265, *p* < 0.001, *df* = 828).

**Table 2:** Differences between older patients with and without PIMs

Patients ≥ 65 years (n = 830)	Without PIM (n = 628)	With PIM (n = 202)	*p*
Age (years)^a^			0.937
65–69	100 (74.6%)	34 (25.4%)	
70–74	176 (77.9%)	50 (22.1%)	
75–79	201 (74.2%)	70 (25.8%)	
80–84	96 (76.2%)	30 (23.8%)	
≥ 85	55 (75.3%)	18 (24.7%)	
Mean ± SD^b^	75.6 ± 5.9	75.6 ± 6.1	0.877
Sex^a^			0.002*
Male	312 (80.6%)	75 (19.4%)	
Female	316 (71.3%)	127 (28.7%)	
Medication plan^a^			0.276
Without plan	196 (74.0%)	69 (26.0%)	
With plan	412 (77.4%)	120 (22.6%)	
Knowledge gaps^a^			0.848
Without knowledge gaps	415 (75.9%)	132 (24.1%)	
With knowledge gaps	213 (75.3%)	70 (24.7%)	
Number of drugs^a^			< 0.001*
< 10	301 (84.8%)	54 (15.2%)	
10–14	251 (73.0%)	93 (27.0%)	
≥ 15	76 (58.0%)	55 (42.0%)	
Mean ± SD^b^	10.2 ± 3.6	12.3 ± 3.8	< 0.001*

*Statistically significant (*p* < 0.05)

^a^Chi squared test was used for categorical variables. Counts and percentages are given

^b^Student’s t-test was used for continuous variables. Means and standard deviations are given

Most of the 247 PIMs were indicated for the nervous system (114), musculoskeletal system (50), cardiovascular system (50) and genitourinary system (19). Seven drugs accounted for half of all PIMs: etoricoxib (29), amitriptyline (22), diazepam (19), dimenhydrinate (14), flecainide (14), doxazosin (13) and solifenacin (13). Notably, 17 PIMs (6.9%) were OTC drugs and another 45 PIMs (18.2%) were prescription (Rx) drugs with sedative and hypnotic properties, i.e. benzodiazepines, zolpidem and zopiclone. One-third of PIMs (n = 81/247) were used as needed.

### Prevalence of drug-related problems

Pharmacists identified DRPs in one-third of medications (n = 3836/11,579) and the majority of patients (n = 918/1090) (Table 3), resulting in 3.5 ± 2.9 DRPs per patient (median: 2, IQR 1–5). Drug-drug-interactions were most frequent, followed by drug use and adherence problems. Pharmacists resolved most DRPs (72.2%) directly with patients, physicians were contacted less frequently.

**Table 3:** Drug-related problems and resolutions

DRP-category (PI-Doc^®^)	DRP frequency		Resolution frequency (drug level)			
	Patient level^a^	Drug level^b^	With patient^c^	With physician^c^	Not possible^c^	Missing data^c^
A: Inappropriate drug choice	197 (18.1%)	332 (2.9%)	221 (66.5%)	60 (18.1%)	16 (4.8%)	35 (10.5%)
C: Inappropriate drug use by patient, including adherence	509 (46.7%)	1043 (9.0%)	841 (80.6%)	110 (10.5%)	27 (2.6%)	65 (6.2%)
D: Inappropriate dosage	208 (19.1%)	304 (2.6%)	207 (68.1%)	66 (21.7%)	14 (4.6%)	17 (5.6%)
W: Drug-drug-interaction	585 (53.7%)	2256 (19.5%)	1566 (69.4%)	305 (13.5%)	131 (5.8%)	254 (11.3%)
U: Adverse drug reaction	231 (21.2%)	379 (3.3%)	276 (72.8%)	62 (16.4%)	20 (5.3%)	21 (5.5%)
S: Other problems	33 (3.0%)	68 (0.6%)	53 (77.9%)	3 (4.4%)	0	12 (17.6%)
Any category	918 (84.2%)	3836 (33.1%)	2769 (72.2%)	488 (12.7%)	192 (5.0%)	387 (10.1%)

Patients and drugs could be affected by multiple drug-related problems of different categories

^a^Percentages refer to the total number of patients (n = 1090)

^b^Percentages refer to the total number of drugs (n = 11,579)

^c^Percentages refer to the number of DRPs in the specified category

**Fig. 1:**
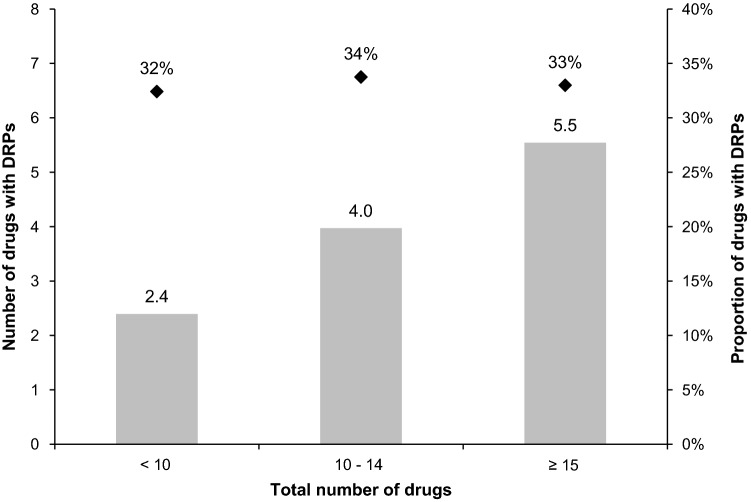
Number and proportion of drugs with DRPs by total number of drugs. The mean number of drugs with DRPs (columns) increased with the total number of drugs, while the proportion (dots) remained equal (n = 1090 patients)

DRPs were more common among Rx drugs (OR 1.634, 95%-CI 1.475–1.812, *p* < 0.001) and medications taken regularly (OR 1.351, 95%-CI 1.218–1.499, *p* < 0.001). The number of drugs with DRPs correlated with the total number of drugs (r = 0.416, *p* < 0.001, *df* = 1088), whereas the proportion remained comparable (Fig. 1).

Pharmacists documented a mean net duration of 66.7 ± 33.7 min per medication review (median: 60, IQR 45–80). Review duration correlated with both DRP and drug numbers (r_(drugs)_ = 0.301, r_(DRPs)_ = 0.270, *p*_(both)_ < 0.001, *df*_(both)_ = 1016).

### Risk factors for drug-related problems

First, we examined potential risk factors for DRPs by bivariate logistic regression analyses (Table 4). DRP risk was increased in patients with knowledge gaps or more drugs, whereas it varied across age groups. Sex and possession of a medication plan did not affect overall DRP risk, however, patients with medication plans had fewer drug use and adherence problems (OR 0.597, 95%-CI 0.462–0.771, *p* < 0.001).

**Table 4:** Risk factors for DRPs among all patients

Independent variable	Bivariate regression analysis			Multivariate regression analysis		
	OR	95%-CI	*p*	OR	95%-CI	*p*
Age						
< 65	1.884	0.986–3.602	0.055	2.888	1.415–5.896	0.004*
65–69	1.427	0.718–2.837	0.311	1.879	0.899–3.930	0.094
70–74	1.672	0.883–3.165	0.115	2.312	1.157–4.621	0.018*
75–79	2.360	1.239–4.498	0.009*	2.764	1.386–5.509	0.004*
80–84	2.098	1.003–4.389	0.049*	2.281	1.049–4.960	0.037*
≥ 85	1 (ref.)			1 (ref.)		
Sex						
Male	1 (ref.)			1 (ref.)		
Female	1.210	0.873–1.678	0.252	1.156	0.806–1.657	0.430
Medication plan						
Without plan	1.161	0.810–1.666	0.416	1.205	0.821–1.767	0.341
With plan	1 (ref.)			1 (ref.)		
Knowledge gaps						
Without knowledge gaps	1 (ref.)			1 (ref.)		
With knowledge gaps	1.917	1.295–2.837	0.001*	1.735	1.129–2.665	0.012*
Number of drugs						
< 10	1 (ref.)			1 (ref.)		
10–14	2.376	1.652–3.418	< 0.001*	2.479	1.658–3.707	< 0.001*
≥ 15	3.450	1.883–6.321	< 0.001*	3.550	1.826–6.902	< 0.001*

*Statistically significant (*p* < 0.05)

Relationships between DRP presence and potential risk factors were analyzed by bivariate logistic regression analysis. All factors were included into multivariate logistic regression analysis, to adjust for confounders. The table depicts results across all patients (n = 1090)

*Ref.* Reference category

Subsequently, we performed multivariate logistic regression analysis to adjust for confounding variables. The influence of age, knowledge gaps and the number of drugs remained statistically significant.

Among older patients, PIM use increased DRP risk significantly in bivariate analysis, but not in multivariate analysis, whereas age, knowledge gaps and the number of drugs remained independent risk factors (Table 5).

**Table 5:** Risk factors for DRPs among older patients

Independent variable	Bivariate regression analysis			Multivariate regression analysis		
	OR	95%-CI	*p*	OR	95%-CI	*p*
Age						
65–69	1.427	0.718–2.837	0.311	1.816	0.863–3.820	0.116
70–74	1.672	0.883–3.165	0.115	2.243	1.117–4.507	0.023*
75–79	2.360	1.239–4.498	0.009*	2.681	1.340–5.366	0.005*
80–84	2.098	1.003–4.389	0.049*	2.249	1.032–4.901	0.041*
≥ 85	1 (ref.)			1 (ref.)		
Sex						
Male	1 (ref.)			1 (ref.)		
Female	1.263	0.868–1.837	0.222	1.172	0.785–1.751	0.437
Medication plan						
Without plan	1.221	0.806–1.851	0.346	1.211	0.785–1.868	0.387
With plan	1 (ref.)			1 (ref.)		
Knowledge gaps						
Without knowledge gaps	1 (ref.)			1 (ref.)		
With knowledge gaps	1.983	1.276–3.082	0.002*	1.721	1.081–2.740	0.022*
Number of drugs						
< 10	1 (ref.)			1 (ref.)		
10–14	2.283	1.512–3.446	< 0.001*	2.186	1.404–3.404	0.001*
≥ 15	4.618	2.167–9.841	< 0.001*	3.827	1.751–8.365	0.001*
PIM use						
No PIM use	1 (ref.)			1 (ref.)		
With PIM use	1.810	1.101–2.975	0.019*	1.377	0.811–2.340	0.236

*Statistically significant (*p* < 0.05)

Relationships between DRP presence and potential risk factors were analyzed by bivariate logistic regression analysis. All factors were included into multivariate logistic regression analysis, to adjust for confounders. The table depicts results for patients ≥ 65 years (n = 830)

*Ref.* Reference category

## Discussion

Pharmacists identified a considerable number of DRPs, suggesting that medication reviews in community pharmacies can promote medication safety. Older patients frequently used PIMs, which indicated a higher DRP risk. However, only knowledge gaps, more drugs and a patient age between 70 and 84 years were independently associated with an increased DRP risk.

### Patient characteristics

With a mean age of 72 years, balanced sex ratio and frequent polypharmacy, our patient sample was comparable to other pharmacy-based studies [22, 28]. Notably, medication plans were common, although not mandatory before 2016. They did, however, not prevent knowledge gaps. While we did not assess completeness of medication plans, other authors found discrepancies in 93% [28]. Knowledge gaps were more common among men and older patients, confirming earlier research [29]. They were less frequent for OTC medications, presumably because these are commonly bought by the patients themselves, with advice from pharmacists.

### Potentially inappropriate medication in older patients

The 24% PIM prevalence we determined resembles results from ambulatory care settings (17–25%) [30, 31], emergency wards (17–36%) [8, 32] and health insurances (19–25%) [33–36], despite methodological differences, such as the absence of daily doses, OTC medications and private prescriptions in insurance data. However, a fourth of the PIMs in our study are OTC medications or commonly prescribed privately, i.e. sedative drugs [37], emphasizing these drugs’ importance for medication safety.

Expectedly, patients with more drugs also used more PIMs. Among women, PIM use was higher as well, caused by more frequent use of psychotropic drugs [38], including common PIMs amitriptyline, diazepam and dimenhydrinate. Both findings confirm results by others [34], who in addition reported a correlation between PIM use and patient age that we cannot confirm. However, their study relied on data from 2007, before the PRISCUS list was published, and awareness about PIM in older patients might have increased since.

Our findings indicate a substantial risk for adverse events, e.g. falls, in older patients. Considering main indications of PIMs, medication safety could benefit particularly from healthcare professionals’ focused attention to drugs for the nervous, musculoskeletal, cardiovascular and genitourinary system.

### Prevalence of drug-related problems

The high number (mean: 3.5, median 2) and prevalence (84%) of DRPs likely results from selective inclusion of high-risk patients by pharmacists. Recruiting similarly, others [22] found an even higher number (5.8) and prevalence (95%) of DRPs and information needs. Conversely, DRPs were found in only 18–21% of consecutively included patients [16, 17]. These differences indicate that pharmacists selected predominantly high-risk patients, where possible. In routine-care-settings, comparable DRP numbers have been found: Dutch community pharmacists identified 3.0 DRPs (median: 2) [39] and Australian pharmacists identified 4.9 DRPs per patient [40], however, those medication reviews incorporated clinical data. Altogether, despite methodological differences, the results endorse pharmacists’ ability to successfully identify problems within their patient’s medication. We consider a focus on patients with maximum intervention benefit essential for the efficient conduction of medication reviews on a larger scale.

As in previous studies [18, 22], drug–drug-interactions were the most frequent DRPs, to which several factors may contribute: First, patients used a high number of drugs, which increases the possibility of drug–drug-interactions exponentially [41]. Second, pharmacy software facilitates the identification of drug–drug-interactions with varying clinical relevance. Finally, DRPs were documented for each drug involved in a drug–drug-interaction. Second-most common were drug use and adherence problems, which were frequently resolved without physicians, emphasizing the proficiency of pharmacists in this area of care. Adverse drug reactions, which were prevalent in one-fifth of patients and involve manifest harmful effects, were resolved in nearly 90% of cases.

According to their documentation, pharmacists resolved most DRPs (72.2%) directly with patients. They contacted physicians in 12.7% of cases, a low proportion compared to previous research [22]. Occasionally, pharmacists might have sent patients to see their physicians about DRPs, which is viable for minor problems and inevitable if patients object to direct contact between healthcare professionals. Thus, some DRPs might have required contact between patient and physician subsequently. Regrettably, follow-up-data was not available. To conclude, although pharmacists identified a considerable proportion of DRPs, physicians’ cooperation is essential to resolve DRPs completely, particularly in the 80% Rx drugs in our sample. On the other hand, 20% of all medications were available over-the-counter. Others [42] found that patients had 2.8 more drugs at home than their physicians’ documentation suggested. Since physicians are frequently unaware of their patients’ OTC drugs, pharmacists are well-positioned for medication reviews incorporating both Rx and OTC medications.

Review duration varied considerably, partly resulting from varying numbers of drugs and DRPs. The remainder might be attributable to differences in pharmacists’ experience, communication, research and documentation. The mean duration of 67 min (median: 60) fits in with other studies (35–90 min) [22, 43]. Variation between studies might reflect differences in review procedure and documentation.

### Risk factors for drug-related problems

The single most important risk factor for DRPs was the number of drugs. It also increased the number of DRPs, while their proportion remained stable, confirming earlier research [22, 44, 45]. Thus, reducing the number of drugs lowers DRP risk as well as PIM exposure. However, deprescribing is challenging and polypharmacy often results from guideline adherence in multimorbid patients [46]. Deprescribing therefore benefits from interdisciplinary collaboration facilitated by systematic medication reviews [47].

Knowledge gaps, which had already been linked to adherence issues by previous research [29], were another risk factor. Surprisingly, medication plans did not affect DRP risk, possibly because they did not prevent knowledge gaps. Consequently, consolidation of patients’ knowledge on drug indications should be considered an integral part of medication reviews.

Among older patients, the risk for DRPs increased with age, likely attributable to multimorbidity and polypharmacy. Notably, it was lower in very old patients, possibly because of elevated awareness of DRPs in frail patients. Interestingly, patients younger than 65 years were at high risk for DRPs. Pharmacists may have included young patients due to their above-average morbidity. Some authors [45] determined a higher DRP risk for patients over 60 years, whereas others [44] found no influence of age. Sex was neither a risk factor in those studies nor in our own research. Older patients with PIMs were more likely to have DRPs as well. However, this association did not persist in adjusted analysis, suggesting confounding variables such as the number of drugs, which was associated with both PIM and DRP prevalence. Although PIMs did not independently increase DRP risk, they indicated potential to optimize medication safety.

### Limitations

Our study had several limitations. First, all data were pharmacist-reported. Varying numbers of DRPs might be partly caused by limited inter-rater-reliability. However, some variation is inevitable in a multi-centered, real-life approach involving hundreds of pharmacists.

Second, this study only included patients who appeared personally and participated in medication anamnesis and discussion. Hence, it excluded other patient groups (e.g. nursing home residents, patients with dementia and immobile patients), for whom transferability of our results might be low. Additionally, patient selection by pharmacists facilitated efficient identification and solution of problems, but limits the generalization of findings.

Third, patients declared that 90% of their drugs were “presently used”. Since this term might be subject to interpretation, particularly for as needed drugs, we included all drugs into analysis. Thus, the numbers of drugs, PIMs and DRPs in use might be up to 10% lower than reported here.

## Conclusion

Pharmacists identified and resolved considerable rates of DRPs. Their assessment provided a viable measure to select patients for review. We conclude that pharmacists are capable and well-positioned to conduct medication reviews and thereby increase medication safety. Several factors were associated with DRPs: Knowledge gaps, the number of drugs, patient age and, in older patients, PIMs may serve as indicators for DRP risk and potential medication review benefit. Thus, systematic pharmacy-based medication reviews can focus awareness and care activities on patients who will benefit most.

Future research should employ the use of standardized tools to ensure reproducible DRP identification and improve inter-rater-reliability, such as lists and algorithms; however, divergent decisions based on patients’ individual needs should remain possible. Additional research is needed on pharmacy-based medication reviews for patient groups not included here, possibly because of immobility, dementia or nursing home residence.
